# Thyroid Hormone Mimetics: the Past, Current Status and Future Challenges

**DOI:** 10.1007/s11883-016-0564-7

**Published:** 2016-02-17

**Authors:** L.P.B. Elbers, J.J.P. Kastelein, B. Sjouke

**Affiliations:** Department of Vascular Medicine, Academic Medical Center, Meibergdreef 9, 1105 AZ Amsterdam, The Netherlands; Department of Internal Medicine, Medical Center Slotervaart, Amsterdam, The Netherlands

**Keywords:** Thyroid hormone mimetics, Thyroid hormone receptor, Dyslipidemia, Low-density lipoprotein cholesterol

## Abstract

The association between thyroid hormone status and plasma levels of low-density lipoprotein cholesterol has raised the awareness for the development of thyroid hormone mimetics as lipid-lowering agents. The discovery of the two main types of thyroid hormone receptors (α and β) as well as the development of novel combinatorial chemistry providing organ specificity has drastically improved the selectivity of these compounds. In the past decades, several thyroid hormone mimetics have been investigated with the purpose of lowering low-density lipoprotein cholesterol levels. However, until now, none of the thyromimetics reached the stage of completing a phase III clinical trial without deleterious side effects. Here, we review the currently available literature on thyromimetics investigated for the treatment of dyslipidemia, their rise, their downfall and the challenges for the development of novel agents.

## Introduction

**Fig. 1:**
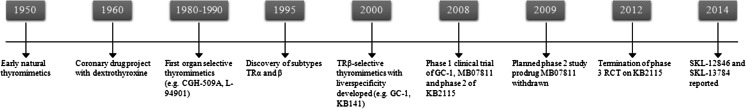
Milestones in the development of thyroid hormone mimetics

Since the 1950s, thyroid hormones have been shown to affect lipid homeostasis [1] and thyroid hormone status has shown to be inversely related to low-density lipoprotein cholesterol (LDL-C) levels. In line, physicians and researchers have appreciated the relationship between hypothyroidism and atherosclerotic vascular disease for over 100 years [2]. Thyroid hormone supplementation results in beneficial effects on lipid and lipoprotein concentrations in patients with hypothyroidism [3], and the American Thyroid Association has recommended that all patients with hypercholesterolaemia should be screened for thyroid dysfunction prior to initiation of lipid-lowering therapy [4]. The association between thyroid hormone status and atherogenic lipoprotein particles has raised the attention for thyroid hormone mimetics as lipid-lowering agents. Although the precise mechanism of atherogenic lipoprotein particle reduction by thyroid hormone and thyroid hormone mimetics is not completely elucidated to date, several mechanisms have been proposed. First, thyroid hormone increases the activity of the promotor of the human low-density lipoprotein receptor (LDLR) gene, resulting in increased LDLR expression and, as a consequence, decreased plasma LDL-C levels [5]. Moreover, thyroid hormone mimetics have shown to induce Cyp7a1, the rate-limiting enzyme of bile acid synthesis, independent of the LDLR, in LDLR knockout mice [6]. Third, thyroid hormone has shown to induce reverse cholesterol transport via upregulation of hepatic scavenger receptor B1 (SR-B1) levels [7]. The discovery of the two main types of thyroid hormone receptors (TRs TRα and TRβ) [8] as well as the development of combinational chemistry to provide organ specificity has drastically improved the selectivity of thyroid hormone mimetics, and some have shown to significantly reduce atherosclerosis in apolipoprotein E (ApoE) knockout mice, an established pre-clinical model for atherosclerosis [9–11]. However, to date, (potential) side effects have limited their clinical use in the arena of cardiometabolic disease. Here, we discuss the different thyromimetics that have been investigated for the treatment of dyslipidemia, their discontinuation and the challenges for the development of novel compounds (Fig. 1). Moreover, we provide a literature update on the thyromimetics currently in development for the treatment of dyslipidemia.

## Thyroid and Thyroid Hormone

One of the first studies that tested the use of thyroid (hormone) to reduce plasma cholesterol in human was published in 1957 [12]. It was observed that administration of dried thyroid reduced plasma LDL-C levels, suggesting that this could be considered as an agent for the prevention of coronary heart disease. A few years later (1960s), the Coronary Drug Project (CDP) was performed to determine whether dextrothyroxine, the d-enantiomer of thyroxine, and other lipid-modifying agents improved survival in men who had suffered from a heart attack [13]. Again, the positive effects of LDL-C lowering were observed but the side effects, particularly related to an excess of adverse cardiovascular outcomes, resulted in the discontinuation of this specific arm of the CDP. This further stimulated the justification to develop thyroid hormone analogues that target the liver without the negative effects on the heart and other extrahepatic organs (e.g. complaints of increased metabolism including excessive sweating). Years later, tiratricol (triiodo-thyroacetic acid) was tested in human but this compound also had deleterious effects on the heart and led to increased bone turnover, discontinuing its pursuit for the treatment of cardiometabolic disease [14].

## First Organ-Selective Thyromimetics

In 1986, the first organ-selective thyromimetic, 3,3-dibromo-3′-pyridazinone-1-thyronine (L-94901), was described [15]. This compound had cholesterol-lowering effects in hypothyroid rats without deleterious effects on the heart. At that time, several similar compounds were developed such as CGH-509A and CGS 23425. CGH-509A reduced cholesterol levels in rodents [16]. CGS 23425 decreased the levels of ApoB-100 [17]. CGS 23425 also increased apolipoprotein A1 levels and LDL-C clearance in rats, without cardiotoxicity [18]. T-0681 reduced the development of atherosclerosis by 80 % in rabbits on a high-cholesterol chow diet [10] and promoted reverse cholesterol transport in mice [19]. Due to unclear reasons, the development of these compounds was not pursued in humans.

## Thyroid Hormone Receptor Beta Agonists

After the first efforts on the development of selective thyroid hormone receptor modulators, cloning of the thyroid receptor led to the identification of two major thyroid receptor subtypes with different tissue distributions throughout the body. The TRα isoform is predominantly present in the brain, heart, and skeletal muscles, whereas TRβ is predominantly present in the liver and also in the brain [20]. Efforts were now focused on the design of several TRβ-selective compounds, characterized by increased binding to TRβ compared with TRα receptors. 2,5-Diiodothyropropionic acid (DITPA) is a thyromimetic compound that binds weakly to both TRα as well as TRβ receptors, but with a modestly higher affinity for TRβ. Approximately 6 months of DITPA therapy resulted in a decrease in total cholesterol (TC) as well as LDL-C levels by ~20 and 30 %, respectively, in patients with congestive heart failure [21, 22]. Similar effects were observed when patients used DITPA as an add-on to statin therapy [22]. Body weight was also reduced [21, 22]. DITPA decreased thyroid-stimulating hormone (TSH) levels without inducing signs or symptoms of hypothyroidism or thyrotoxicosis. However, high rates of side effects, including fatigue and gastrointestinal complaints, were observed. Moreover, DITPA therapy resulted in potentially deleterious effects on serum markers of both bone formation (osteocalcin) as well as turnover/degradation (*N*-telopeptide and deoxypyridinoline). As a consequence, the DITPA program was discontinued [21, 22].

GC-1 (or sobetirome; Table 1) is one of the first compounds designed in a series of analogues, with, amongst others, a 3′-isopropyl substitution at the distal phenyl ring of the molecule (instead of iodine in T_3_). GC-1 has at least a ~3–18-fold selectivity for TRβ over TRα [16, 27]. This selectivity is (partly) based on the presence of a single amino acid (Asn-331) within the TRβ domain [27]. Sobetirome has shown to reduce serum cholesterol and triglyceride levels by 25 and 75 %, respectively, in chow-fed euthyroid mice [7]. In a phase I study, GC-1 reduced LDL-C levels up to 41 %, in normolipidaemic subjects, after 2 weeks [23]. In line, Kannisto and co-workers recently showed that GC-1 is able to reduce atherosclerosis, defined as cholesterol content in the arterial wall, in aortas of ApoE-deficient mice [11]. Moreover, in a recent study, it has been shown that unlike 3,5,3′5′-tetraiodothyronine (T_4_), GC-1 did not influence tolerance to physical exercise in hypothyroid rats [28]. This is of importance since both hypothyroidism as well as hyperthyroidism itself are associated with exercise intolerance [29].

**Table 1:** Characteristics of thyromimetics sobetirome, eprotirome and MGL-3196

Compound	Company	Chemical characteristics	Stage of clinical development	Effect on lipid profile	Side effects
Sobetirome (GC-1)	QuatRx	3′-Isopropyl substitution at the distal phenyl ring of the molecule (instead of iodine in T_3_) At least ~3–18-fold selectivity for TRβ over TRα	Terminated after phase I due to serious effects in a similar but not identical compound (eprotirome)	↓ LDL-C up to 41 % [23]	NA, generally well tolerated
Eprotirome (KB2115)	Karo Bio	Modestly higher affinity for TRβ compared to TRα Minimal uptake in non-hepatic tissues compared with T_3_	Terminated during phase III clinical study in patients with familial hypercholesterolaemia	↓ TC by 17–27 % ↓ LDL-C by 22–32 % ↓ TG by 16–33 % ↓ Lp(a) by 27–43 % ↓ ApoB by 21–31 % [24, 25]	Significant increases in transaminase levels in phase III Deleterious effects on cartilage in canines
MGL-3196	Madrigal Pharmaceuticals	Pyridazinone analogue with ~28-fold TRβ selectivity over TRα	Phase I; results of dose interaction study (NCT02542969) are awaited	↓ TC up to 23 % ↓ LDL-C up to 30 % ↓ TG up to 24 % ↓ ApoB up to 60 % [26••]	No evidence for any deleterious effects on the heart and liver, to date

↓ = lowering

*TC* total cholesterol, *LDL-C* low-density lipoprotein cholesterol, *TG* triglycerides, *Lp(a)* lipoprotein (a), *ApoB* apolipoprotein B, *NA* not available

GC-24 is a TRβ receptor agonist with similar affinity for TRβ to TRα, but with a much higher selectivity. This increased selectivity (~40-fold for TRβ over TRα) was reached by the addition of a phenyl group at the 3′ position of the distal aryl ring of GC-1 [28] and the subsequent creation of a new hydrophobic cluster [27]. GC-24, in contrast to GC-1, has shown to have no activity in the brain, which has been suggested to be caused by a limited entry through the blood-brain barrier [30].

Another group of TRβ analogues comprised KB141 and KB2115 (or eprotirome; Table 1). KB141 was designed in a series of compounds varying in length of a carboxylic acid chain, which resulted in a profound effect on affinity and specificity of the agents [31]. Seven days of treatment with 154, 462 or 924 nmol/kg/day of KB-141 resulted in plasma cholesterol reductions up to ~35 % from baseline. Although a small increase in heart rate was observed in cholesterol-fed Sprague Dawley rats treated with KB141, no tachycardia was observed in monkeys [31]. In addition to the identification of the different TR subtypes, the insight that TRα and TRβ are differently distributed throughout the body resulted in the development of agents with both TRβ as well as liver selectivity. Eprotirome, a compound with these characteristics, was the first thyroid hormone mimetic designed for the treatment of dyslipidemia that reached phase III of clinical development. A 12-week treatment with eprotirome as an add-on to statins has shown to significantly decrease LDL-C, triglyceride and lipoprotein (a) levels by 22–32, 16–33 and 27–43 %, respectively, in patients with hypercholesterolaemia [24, 25]. The eprotirome program has, however, been terminated prematurely due to deleterious effects on cartilage observed in canines [32]. A phase III study in patients with familial hypercholesterolaemia, which was performed in parallel with the study in dogs, revealed significant increases in aspartate aminotransferase (AST) and alanine aminotransferase (ALT) levels by 114 and 189 %, respectively, after 6 weeks of treatment with 100 μg of eprotirome [33•]. To date, it is unknown whether these adverse hepatic effects of eprotirome were off-target and compound-specific, due to an effect mimicking thyrotoxicosis in the liver or due to a drug-drug interaction at the level of the liver.

Although an extensive review of the literature about the effects of thyromimetics on hepatic steatosis and insulin sensitivity is beyond the scope of this review, it is worth noting that given the weight-reducing potential of TR activation, both sobetirome as well as eprotirome have been studied as therapeutic strategies for the treatment of metabolic disorders, including non-alcoholic fatty liver disease (NAFLD), in rodents. Although treatment with both GC-1 as well as KB2115 resulted in a decrease of hepatic steatosis, the effects on glycaemia and insulin sensitivity were variable and time-, dosage- and agent-dependent [34, 35].

In the search for finding thyromimetics with selectivity for both TRβ as well as the liver, Madrigal Pharmaceuticals recently developed a series of pyridazinone analogues, which, amongst others, resulted in the identification of MGL-3196 (Table 1). This compound has a 28-fold TRβ selectivity over TRα [36] and is currently being investigated in phase I clinical trials. MGL-3196 significantly reduced LDL-C, ApoB and non-HDL levels up to 30, 24 and 28 %, respectively, after a 2-week daily dose of 5–200 mg (compared with increases of 3.1, 4.2 and 8.9 %, respectively, with placebo). In contrast to eprotirome, which mildly increased transaminase levels in phases 1 and 2 [25, 37], no increases in liver parameters were observed in healthy volunteers with mildly elevated LDL-C levels treated with MGL-3196 for 2 weeks [26••].

Moreover, no evidence for any deleterious effects on the heart was observed [26••]. The question remains whether MGL-3196 is safe as an add-on to statins, since these are considered as the cornerstone for lipid-lowering therapy in patients with dyslipidemia. A phase I dose interaction study (NCT02542969) has recently been completed [38]. The results of this study are eagerly awaited.

## Thyroid Hormone Receptor Beta and Liver-Selective Prodrugs

The goal of the development of liver-targeted prodrugs was to deliver the thyromimetics to the site where cholesterol is produced (i.e. the liver) while reducing the exposure of the compound to other tissues in order to prevent side effects. The liver-selective, cytochrome P450-activated, prodrug MB07811 (2*R*,4*S*)-4-(3-chlorophenyl)-2-[(3,5-dimethyl-4-(4′-hydroxy-3′-isopropylbenzyl)phenoxy)methyl]-2-oxido-[1,3,2]-dioxaphosphonane undergoes first-pass hepatic extraction. Subsequent cleavage of this prodrug generates the negatively charged phosphonate-containing thyromimetic (3,5-dimethyl-4-(4′-hydroxy-3′-isopropylbenzyl)phenoxy)methylphosphonic acid (MB07344), which distributes poorly into most tissues. MB07344 is rapidly eliminated in the bile to escape the enterohepatic recirculation [39] and has been shown to reduce cholesterol and both serum as well as hepatic triglycerides in rats [40]. Other studies showed that MB07811 markedly reduced hepatic steatosis and plasma free fatty acids and triglycerides in rodents with hepatic steatosis [41]. Transaminase levels remained unchanged, and MB07811 did not increase heart weight or decrease pituitary expression of thyroid-stimulating hormone β (TSHβ). Beside this, no effects on muscle and bone were observed at therapeutic dosages [39]. In rabbits, dogs and monkeys, it was observed that the effects of MB07811 and atorvastatin in lowering plasma TC were additive [42]. This led to the hypothesis that this compound could have clinical utility as a treatment to further reduce plasma TC levels in patients that did not yet reach their cholesterol treatment goals despite statin treatment. In 2006, a phase Ia clinical trial demonstrated the safety and tolerability of MB07811 in a single-dose study [43]. The results of a subsequent phase Ib trial with MB07811 were promising since it was both efficacious (placebo-corrected decreases in LDL cholesterol of 15–41 % and in triglyceride levels of >30 % in patients with mild hypercholesterolaemia compared with placebo) as well as safe in different dosages up to 40 mg [44]. No differences in heart rate, heart rhythm or blood pressure, between MB07811- and placebo-treated patients, were observed. Unexpectedly, MB07811 caused a mild elevation of liver enzymes. Beside this, it decreased total and free thyroxine (FT_4_) levels by day 7 with both doses of MB07811 [40]. A phase II randomized, placebo-controlled study assessing the efficacy, safety and tolerability of MB07811 given orally to subjects with primary hypercholesterolaemia for 12 weeks was planned, but this study has been stopped prior to initiation as the developing company Metabasis Therapeutics, Inc. was acquired by Ligand Pharmaceuticals, Inc. [43]. Further trials were not initiated.

## Liver-Selective 1-Benzyl-4-Aminoindole-Based Thyroid Hormone Receptor Beta Agonists

Recently, a series of 1-benzylindole-based TRβ agonists were prepared and evaluated, in a search for more TRβ-selective hepato-specific modulators [45]. This work investigated the potential use of indoles as inner ring isosteres. Two compounds of interest were found, later named as SKL-12846 and SKL-13784. Liver concentrations of these compounds were 100-fold greater than the heart or brain concentrations and at least 10-fold greater than the plasma concentration. The liver specificity of SKL-12846 and SKL-13784 is achieved by active uptake by specific transporters [46]. These two compounds were orally administered to cholesterol-fed rats and showed to produce a significant reduction in TC levels. Of note, heart rate and heart weight increased following treatment with both compounds. The increase in heart rate produced by the two analogues was, however, less than 15 %, which is considered to be the upper limit for clinical use [45]. No effect was seen on TSH levels due to its low brain penetration. A more recent study, however, showed that SKL-13784 significantly reduced endogenous T_4_ levels at doses lower than its lipid-lowering dose, by an unclarified mechanism [47]. This may raise concern over this compound’s ability to alter thyroid hormone metabolism in the liver and, therefore, the impact on the potential usefulness of this liver-selective TRβ agonist. Beside this, the research that has been performed on these compounds, to date, does not rule out whether these compounds could have deleterious effects on the liver itself.

## Conclusion

In summary, during the past decades, several thyromimetics have been developed with varying but convincing efficacies on atherogenic lipids and lipoproteins. Until now, none of the thyromimetics reached the stage of completing a phase III clinical trial without deleterious side effects. Several explanations could underlie these discontinuations. First, the development of TRβ-selective thyromimetics is complicated by the fact that endocrine physiology is highly complex and that the precise distribution of TRα and TRβ throughout the body is not completely elucidated to date. Even if thyroid hormone mimetics were shown not to interfere with the hypothalamic-pituitary-thyroid axis, they could still result in unexpected side effects. Moreover, even if exclusive TRβ and liver selectivity would be reached, this would not exclude a potential effect on other organ systems. It was, for example, recently shown that hyperthyroidism leads to a hypercoagulable state [48] and that patients with hyperthyroidism are at increased risk of developing venous thrombosis [49–51]. One could speculate that the agonistic effect of thyromimetics on the TRβ could induce a hypercoagulable state.

To the best of our knowledge, currently, only MGL-3196 is being actively tested in humans. The effects of MGL-3196, resulting in LDL-C reductions up to 30 % from baseline without effects on liver parameters, are promising, and the results of recently completed clinical trials (e.g. NCT02542969) and the effects on cardiovascular outcomes need to be awaited.
